# The Role of the Polyethylene Glycol in the Organization of Gold Nanorods at the Air–Water and Air–Solid Interfaces

**DOI:** 10.1021/acs.langmuir.4c01427

**Published:** 2024-07-04

**Authors:** Michał Kotkowiak, Beata Tim, Mateusz Kotkowiak, Joanna Musiał, Paulina Błaszkiewicz

**Affiliations:** † Faculty of Materials Engineering and Technical Physics, Poznan University of Technology, Piotrowo 3, 60-965 Poznan, Poland; ‡ Department of Rare Earths, Faculty of Chemistry, Adam Mickiewicz University, 61-614 Poznan, Poland

## Abstract

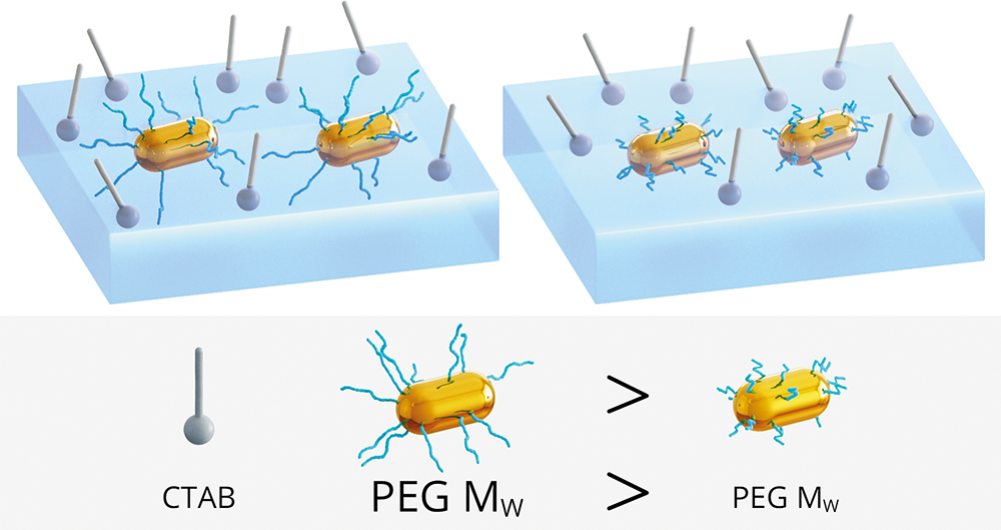

The organization of metallic nanoparticles into assembled films is a complex process. The type of nanoparticle stabilizing ligand and the method for creating an organized layer can profoundly affect the optical properties of the resulting nanoparticle assembly. Investigations of the ligand structure and nanoparticle interactions can provide a greater understanding of the design of the assembly process and the quality of the resulting materials. One of the functionalization methods in the preparation of specific gold nanorods is the utilization of thiol-terminated poly(ethylene glycol). This generates gold nanorods capable of forming stable monolayers at the air–water interface upon dispersion in a suitable organic solvent. Herein, we show that depending on the molecular weight of the poly(ethylene glycol), the structures obtained at the air–water and air–solid interfaces differ in the arrangement. The studied structures were characterized by using spectroscopic and microscopic techniques, and the structural type was correlated with the polymer type. Insoluble and stable Langmuir monolayers composed of higher-molecular-weight gold nanorods with poly(ethylene glycol) were formed only in the presence of an additional stabilizer that prevented the formation of gold nanorods in aqueous solutions. At the air–solid interface, conformational changes in poly(ethylene glycol) induced the aggregation of gold nanorods, which became closely packed under the influence of surface pressure. The presented results suggested that the arrangement of two-dimensional layers of gold nanorods could be tailored using poly(ethylene glycol) of various molecular weights.

## Introduction

Gold nanoparticles (Au-NPs) exhibit excellent physical, chemical, and biological properties; therefore, they have potential applications in many areas of technology.^1−3^ The most crucial feature of NPs that benefits their applications is localized surface plasmon resonance (LSPR). Illumination of NPs induces charge oscillation and produces local electric field enhancement, which enhances photothermal conversion.^4^ Depending on the preparation conditions, NPs with different shapes exhibiting LSPR in various spectral regions can be obtained.^5^

Spectroscopic studies of gold nanorods (Au-NRs) have two LSPRs in the transverse and longitudinal directions. The longitudinal LSPR is tunable in the spectral range from visible to near-infrared.^6^ Au-NRs change their shape on the spherical surface under intense laser exposure. However, this depends on the particle’s surfaces. Hence, appropriate functionalization should prevent shape deformation upon exposure to light. Additionally, control of the surface functionalization of Au-NRs is necessary for NPs to find many applications in various fields. However, difficulties arise in the exchange of the standard surface ligands, and the typical stabilizer of Au-NRs, cetyltrimethylammonium bromide (CTAB), is added, making it more challenging to promote the exchange in spherical NPs compared to sodium citrate.^7^ In contrast to sodium citrate, CTAB forms a densely packed bilayer at the Au-NRs surface, and to reach the necessary stability, unbound CTAB in Au-NRs colloidal dispersion is required. Considering the toxicity of CTAB, the CTAB bilayer of Au-NRs was liganded with thiolated poly(ethylene glycol) (PEG). To reduce the cytotoxicity, Au-NRs were washed by centrifugation. However, the CTAB bilayer tends to remain on the surface of the Au-NRs, and CTAB bilayers are not covalently adsorbed on the surface. This suggests that further removal of CTAB leads to aggregation of the Au-NRs.^8^ PEG was used as a linker for Au-NRs to facilitate the attachment of other functional groups to the surface of the Au-NRs via Au–S bonding. Due to its high affinity for gold, its biocompatibility, and its key role in improving the colloidal stability and dispersibility of Au-NRs in aqueous media, PEG is most commonly used in ligand exchange reactions with Au-NRs.^9^ The basic method of Au-NRs PEGylation involves one-step ligand exchange. Potassium carbonate and PEG are added directly to the Au-NRs solution. Other PEGylation protocols consider the influence of pH; e.g., potassium carbonate was added under alkaline conditions. However, the use of an acidic environment at approximately pH of 3 has been reported as well.^10^ PEGylation is widely applied as a surface modification method for NPs in biomedical applications to improve their biological properties, including biocompatibility and immunogenicity. The functionalization of Au-NRs with thiol-terminated PEGs yields PEGylated Au-NRs with enhanced stability and biocompatibility. The retention time of such Au-NRs in an aqueous medium is prolonged. Furthermore, PEGylated Au-NRs disperse in aqueous and several organic polar mediums, such as acetone, alcohols, acetonitrile, dimethyl sulfoxide, dimethylformamide, and phosphate-buffered saline solutions.^11^ Therefore, PEGylated Au-NRs dispersed in a suitable organic solvent, e.g., chloroform, form stable monolayers at the air–water interface.^12^

The Langmuir technique is used to study nanoparticles and more complex systems, including proteins or lipids.^13^ Monolayers containing NPs can be the basis for producing so-called nanoplatforms that can enhance Raman intensity. For this purpose, the monolayers produced by the Langmuir technique can be transferred to a solid substrate using the Langmuir–Blodgett (LB) or Langmuir–Schaefer (LS) techniques.^14,15^ In our recent publication,^12^ we described dendritic, branched structures of Au-NRs upon LB extraction and their application in the amplified detection of molecules. PEG-2k was selected to ensure a thick layer formation on the surface of Au-NRs. It was found that the thinner the layer, the more hot spots that could be induced. PEG coating is also a crucial factor in improving the biophysical properties of NPs, which is important for drug delivery systems.^16,17^

Herein, the physicochemical problem of the Au-NRs pattern formation was explored in greater detail. The PEG type in the organization of Au-NRs at the air–water and air–solid interfaces was examined. The type of formed structures was carefully characterized by using spectroscopic and microscopic techniques. Additionally, this was related to the PEG chain conformation type.

## Materials and Methods

### Chemicals

Tetrachloroauric acid (HAuCl_4_·H_2_O) (99.99%) was obtained from Alfa Aesar. Cetyltrimethylammonium bromide (CTAB) (99.00%), sodium borohydride (NaBH_4_) (98.00%), silver nitrate (AgNO_3_) (99.99%), ascorbic acid (99.00%), *O*-(2-mercaptoethyl)-*O*′-methylpoly(ethylene glycol) (*M*_w_ ≈ 2000; PEG-2k) (99.99%), *O*-[2-(3-mercaptopropionylamino)ethyl]-*O*′-methylpoly(ethylene glycol) (*M*_w_ ≈ 5000; PEG-5k) (99.99%), and *O*-(2-mercaptoethyl)-O′-methylpoly(ethylene glycol) (*M*_w_ ≈ 10,000; PEG-10k) (99.99%) were purchased from Sigma-Aldrich. Spectrophotometric grade methanol was purchased from POCH S.A. (Poland). High-purity chloroform for spectroscopy (CHCl_3_) (>99% Uvasol), isopropanol (≥99.8%), and acetone (≥99.8%) were purchased from Merck.

### Chemical Synthesis of Rod-Shaped Gold Nanoparticles and Functionalization by Polymer Coating

Aqua regia (HCl: HNO_3_ 3:1 (v/v)) was used to treat the glass before the synthesis of NPs. The process utilized ultrapure water (Milli-Q, 18.2 MΩ·cm, 71.98 ± 0.01 mN·m^–1^). Au-NRs were prepared following the procedure described by Nikoobakht et al.^18^ with the modifications previously described by Błaszkiewicz et al.^19^ The Au-NRs were functionalized with PEG using a modified method.^12,20^ Au-NRs with a maximum absorption wavelength in the longitudinal band around 680 nm were used for the functionalization process. Their size is length (53.2 ± 1.8) nm and width (23.6 ± 1.3) nm.^19^ To experimentally determine the concentration of PEGylated Au-NRs, we used inductively coupled plasma optical emission spectroscopy (ICP-OES). For this purpose, we determined the gold concentration and the gold volume per particle from the TEM measurement of particle dimensions, assuming their cylindrical shape. Dividing these numbers gave us the particle concentration. The concentrations of gold were as follows: 65 ± 4, 66 ± 4 and 61 ± 4 mg·L^–1^ for Au-NRs PEG-2k, 5k and 10k, respectively. The Au-NRs concentration was 7.2 × 10^–10^, 7.4 × 10^–10^, and 6.7 × 10^–10^ M for Au-NRs PEG-2k, 5k, and 10k, respectively. Au-NRs were centrifuged twice for 30 min to remove excess CTAB. Then, 10 mL of 1 mM PEG (2k, 5k, and 10k) solution was prepared and sonicated for 30 min. The centrifuged Au-NRs were redispersed in PEG solution and left stirring for 24 h at room temperature. Subsequently, the unbound PEG molecules were removed by centrifugation (twice for 30 min). The supernatant was discarded, and the Au-NRs pellet was dispersed in methanol. The functionalization reaction scheme is shown in Figure S1 (refer to the Supporting Information).

### Surface Film Preparation

To obtain monolayers at the air–water interface, chloroform Au-NRs dispersions were used, according to the method described by Tim et al.^12^ In the prepared mixtures of Au-NRs with chloroform, the ratio of methanol to chloroform was 1:4 (v/v). The surface films produced by the Langmuir technique were then transferred to the solid substrates (quartz plates) in two ways: using the LB and LS techniques. At the beginning of each experiment, the Langmuir trough (KSV Nima) (304 mm × 75 mm) was filled with ultrapure water, which was the subphase. Then, Au-NR dispersions were spread on the surface of the subphase and the solvent was evaporated (20 min). The layer was compressed by symmetrical movement of two Delrin polymer barriers at a constant speed of 5 mm·min^–1^. Changes in the surface pressure (π) values during film formation were measured using a platinum Wilhelmy plate combined with a computer-controlled Langmuir balance (KSV Nima, instrument precision of 0.01 mN·m^–1^). The Langmuir balance was equipped with a Brewster angle microscope (MicroBAM, KSV Nima), and the structural changes occurring during monolayer compression were recorded. The stability of the monolayer was measured using the Langmuir technique by performing relaxation experiments. For this purpose, the monolayer from Au-NRs dispersion with PEG-2k brushes was compressed (with a constant speed of 5 mm·min^–1^) to π equal to 4, 8, 15, 23, and 37 mN·m^–1^, and then the change of the relative surface area (A·A_0_^–1^) over time (t) was recorded. In the next stage, the specific π for which relaxation experiments were performed, the monolayers were transferred to solid substrates by using LB and LS techniques. For this purpose, Au-NRs with different PEGs (2k, 5k, and 10k) were employed. Before the experiment, quartz plates (30 mm × 25 mm × 1 mm) were cleaned using an ultrasound bath for 20 min at a temperature of 70 °C in a mixture of ultrapure water, 4% ammonia, and 5% hydrogen peroxide and then rinsed in ultrapure water. In the case of the LB technique, the speed was 3 mm·min^–1^ for the upstroke. However, for the LS technique, it was 0.5 mm·min^–1^. During Langmuir monolayer deposition, the transfer ratio (TR) was monitored. For LB, the TR was around 1 in all of the analyzed cases. It should be noted that reliable and repeatable TR values are difficult to obtain for LS deposition. The operating software KSV Nima provided TR values. However, due to the relatively large dimensions of the substrate, the TR value could be overestimated using the LS technique. Thus, visual observation was prioritized during the transference to ensure that the transferred material was deposited uniformly. Each experiment was carried out in triplicate to ensure the repeatability of the curves at a constant temperature of 21 ± 1 °C.

### Microscopic and Spectroscopic Measurements

The morphology of Au-NRs layers deposited on quartz substrates at different π values was determined using confocal laser scanning microscopy (LSM710, Zeiss, Germany). In the material mode (reflected light), the He–Ne laser was operated at a wavelength of 543 nm. Images were collected from Z planes using a Z Stack Module to acquire Z-stacks with a motorized focus drive. ImageJ processing software was used to calculate the surface coverage and standard deviation. For this purpose, 10 independent images of the obtained layers were considered. Images were analyzed by using the main thresholding command in ImageJ. Electronic absorption spectra of the Au-NRs layers were measured by using a Varian Cary 4000 spectrometer. The Tescan Mira 3 scanning electron microscope (SEM) was used to investigate the Langmuir–Blodgett Au-NRs layers. Because of the thin Au layer, the acceleration voltage was equal to 12 kV. This investigation used secondary electron contrast to improve the image collection.

### Dynamic Laser Scattering Measurements

The hydrodynamic diameters (HD) and the zeta potential of the PEG-coated Au-NRs were determined at 25 °C using a Malvern Zetasizer Nano ZS equipped with a laser of 632.8 nm wavelength in backward scattering mode (173°).

## Results and Discussion

The absorption spectra of Au-NRs solutions, TEM images, and DLS measurement analysis are shown in Figures S2–S4 and Table S1. The LSPR band positions of PEGylated nanorods were almost the same as those of unmodified Au-NRs. No apparent shifts were observed for the LSPR peaks (see Table S1), and a very small red shift was detected for the peaks after PEGylation. Both the red shift and blue shift of the LSPR peak have been reported after PEGylation of Au-NRs.^10^

The size of the CTAB-protected and PEGylated Au-NRs was determined using TEM and DLS. TEM micrographs showed the nanorod shape and the nanoscale size of the CTAB-protected materials (Figure S3). The longitudinal dimensions of the nanorods were from 20 to 60 nm. HD and zeta potential measurements were carried out using a dynamic laser scattering technique. Size distribution report by intensity showed two peaks (Figure S4). The peak localized at the lower size range, a few nanometers, is sometimes mistaken as the presence of small particle impurities. However, it was recently shown that it can correspond to the rotational diffusion of the nonspherical Au-NRs and should not be considered an actual particle size distribution peak.^21^ This peak signifies that the rotational diffusion coefficient of the Au-NRs is equivalent to the translational diffusion coefficient of a spherical particle. The other peak, located at the higher size range, corresponds to the actual size of the solvated PEG-functionalized Au-NRs. The mean HD of PEG-2k, PEG-5k, and PEG-10k-functionalized Au-NRs was equal to 68 ± 2, 84 ± 3, and 72 ± 2 nm, respectively (Table S1). These values are in agreement with the size of CTAB-protected Au-NRs particles determined using TEM. It is important to note that DLS determines the nanoparticle size by assessing the particle’s diffusion coefficient. The measurement result is HD representing the size of a solvated particle, including its electrical double layer. In addition, the diffusion coefficient is not solely influenced by the particle’s mass; factors such as shape and surface chemistry also play a role.

The zeta potential of the CTAB-protected Au-NRs was equal to 34 mV, which confirms their good stability in an aqueous environment (Table S1). The positive charge stems from the cationic nature of CTAB, which, as a surfactant, forms a bilayer surrounding the nanorod. The PEGylation consisted of replacing the CTAB bilayer with PEG chains attached to the Au-NRs surface via the thiol moiety. The replacement of CTAB by thiol-PEG can be confirmed using Raman spectroscopy by registering the appearance of the Au–S band and the disappearance of the Au–Br band, which was carried out in our earlier work.^10,12^ The zeta potential measured for 2, 5, and 10,000 PEG-functionalized Au-NRs was equal to 15.0, 11.4, and −10.6 mV, respectively (Table S1). These values represent the charge shielding ability of different PEG chain lengths and can be explained as follows. Upon the replacement of the positively charged CTAB bilayer by 2k or 5k PEG layer, the negative charge of the bare Au-NR is screened by neutral—and relatively short, compared to 10k PEG—loose polymer chains. However, some positively charged ions can still be attracted by the negatively charged Au-NRs and tend to form positively charged Stern and diffusion layers around the particle. In the case of 10k PEG, the polymer chains can provide a more compact shield of the Au-NRs, and the positively charged ions no longer tend to approach the particle surface. PEG molecules have a neutral to slightly negative surface charge, which may be the reason for the negative zeta potential values of the 10k PEG-functionalized Au-NRs. These results are in accordance with the literature.^8,10^ Noteworthy, in addition to the attachment of the PEG chain to the Au-NR surface via the −SH moiety and electrostatic repulsion between the particles, PEG also provides steric stabilization of the materials. Sufficient PEG coating thickness prevents the aggregation of the materials caused by the van der Waals interactions and ensures good dispersion of the particles in the system.

CTAB plays many roles in Au-NRs synthesis,^22^ including the organization of Au-NRs and the air–water and air–solid interfaces. The amount of CTAB in a spreading solution influences the shape of the Langmuir monolayer isotherm and its stability over time, which was previously discussed^12^ and described in this work. A stable dispersion was obtained by developing a spreading solvent mixture (see the Experimental Section for details)^12^ to adjust the hydrophilic character of the Au-NRs. However, for higher mass PEGs, the Langmuir monolayers should be obtained in the presence of excess CTAB, which stabilizes Au-NRs functionalized with PEG-5k and PEG-10k (results not shown). To quantify the amount of CTAB spread at the air–water interface, the procedure reported by Adura et al.^23^ provided sufficient and reliable results.^12,24^ Three-time centrifugation caused the aggregation of Au-NRs when CTAB concentration in solution was (3.3 ± 0.4) × 10^–6^ M. The CTAB concentration that remained in the spreading solution was three times higher than that of PEG-2k Au-NRs.^12^ Higher concentrations of CTAB have a positive influence on the Langmuir monolayers of Au-NRs. The increased CTAB content promoted stability in the Langmuir monolayer over the entire π range for PEG-2k (Figure S5), and the same effect was observed in the rest of the PEGs (results not shown). In our recent work on diketopyrrolopyrroles (DPPs)/4-octyl-4′-cyanobiphenyl (8CB) mixtures, the stability was significantly improved over time. The confocal microscopy investigation revealed almost complete removal of agglomerate structures in the DPP with 8CB.^25^

**Figure 1:**
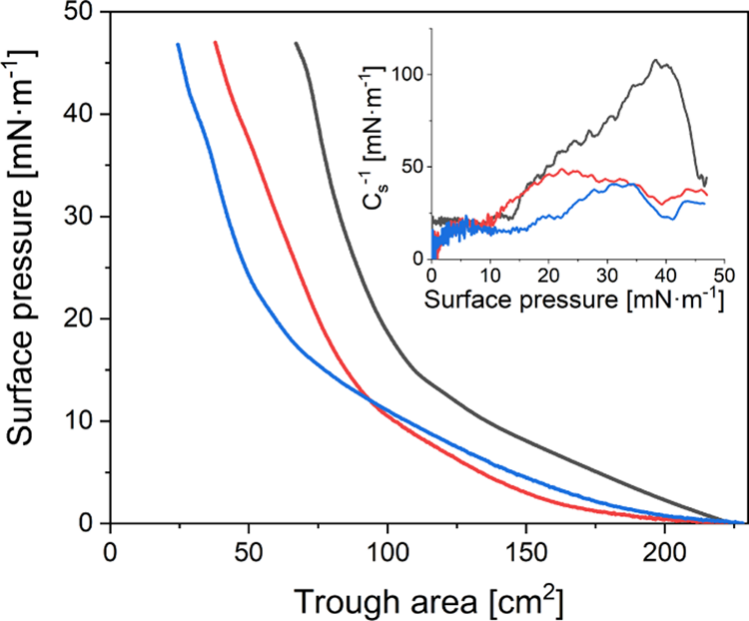
Surface pressure vs trough area isotherm of Langmuir monolayer for gold nanorods for different PEG: PEG-2k (black line), PEG-5k (red line), and PEG-10k (blue line); the inset graph shows the dependence of the surface compressional modulus *C*_s_^–1^ on the surface pressure of Au-NRs.

Compared to previous reports, the obtained Langmuir monolayers were more stable, as shown in Figure S5.^12^ The deposition process could be more precisely and easily controlled. In this work, a higher value of CTAB was carefully selected according to the centrifugation procedure. The confirmation when using a higher amount of CTAB was also recorded for the π–*A* isotherm for the PEG-2k monolayer (Figure 1). In this case, higher values of π were obtained for the maximum value of surface film compression compared to published studies.^12^

Measurements of π–*A* isotherms carried out during the compression of the monolayers consisting of Au-NRs with different amounts of PEG allowed us to determine the effect of PEG chain length on the thermodynamic properties of the surface films. An increase in the molecular weight of PEG resulted in a shift of the π–*A* isotherm toward lower values of the trough area. Additionally, the PEG footprint increased while PEG grafting density decreased.^7^ Thus, excess PEG molecules used during Au-NRs functionalization promoted the attachment of more PEG of lower molecular weight to the Au-NRs surface. Therefore, an alkyl chain of higher molecular weight was used to be more bent than the shorter one. In the case of PEG-2k, the increase in π occurred immediately after compression was started. However, for PEG-5k and PEG-10k, it occurred at the surface of the trough of 195 cm^2^. Depending on the molecular weight of PEG, slight differences were observed during the course of the isotherms. For the PEG-2k, PEG-5k, and PEG-10k isotherms, the value of π increased gradually to 14, 11, and 18 mN·m^–1^, respectively. Above these values, the compression of the monolayers showed a more rapid increase in the π value.

Based on π–*A* isotherms, the compression modulus values (*C*_s_^–1^) were calculated, which was defined as . The *C*_s_^–1^ parameter, introduced by Davies and Rideal, allowed us to determine the physical state, elasticity, and/or packing changes in a monolayer.^26^ The obtained ranges of the *C*_s_^–1^ modulus corresponded to the physical states of the monolayer. The studied monolayer was considered to be in the gaseous state (G) if the *C*_s_^–1^ values were below 12.5 mN·m^–1^. However, the state in the liquid (LE) and condensed in the liquid (LC) had *C*_s_^–1^ values in the ranges between 12.5–50 and 50–250 mN·m^–1^, respectively. Above 250 mN·m^–1^, the monolayer was assumed to be in the solid phase (S).^26^ Analysis of the dependence of the compressibility modulus *C*_s_^–1^ as a function of π, shown in Figure 1, proved that the molecular weight of PEG also affected the elasticity of the monolayers. For PEG-2k, the maximum value of *C*_s_^–1^ was 108 mN·m^–1^, which corresponded to the LC phase and was characterized by reduced flexibility compared with monolayers consisting of Au-NRs coated with PEG with higher molecular weights. In the case of PEG-5k and PEG-10k, the formed films were determined as the LE phase because the maximum values of the compressibility modulus *C*_s_^–1^ were 49 and 40 mN·m^–1^, respectively.

In the presence of PEG with a higher molecular weight, the ligand density decreased due to the larger PEG molecules that consumed more space and surface area around the anchoring thiol group. This, in turn, reduced the number of PEG molecules needed to saturate the surface and achieve a stable dispersion. Thus, when using PEG of decreasing length, a high amount of PEG was required to achieve stable PEGylated NPs and a critical stability ratio.^27^

**Figure 2:**
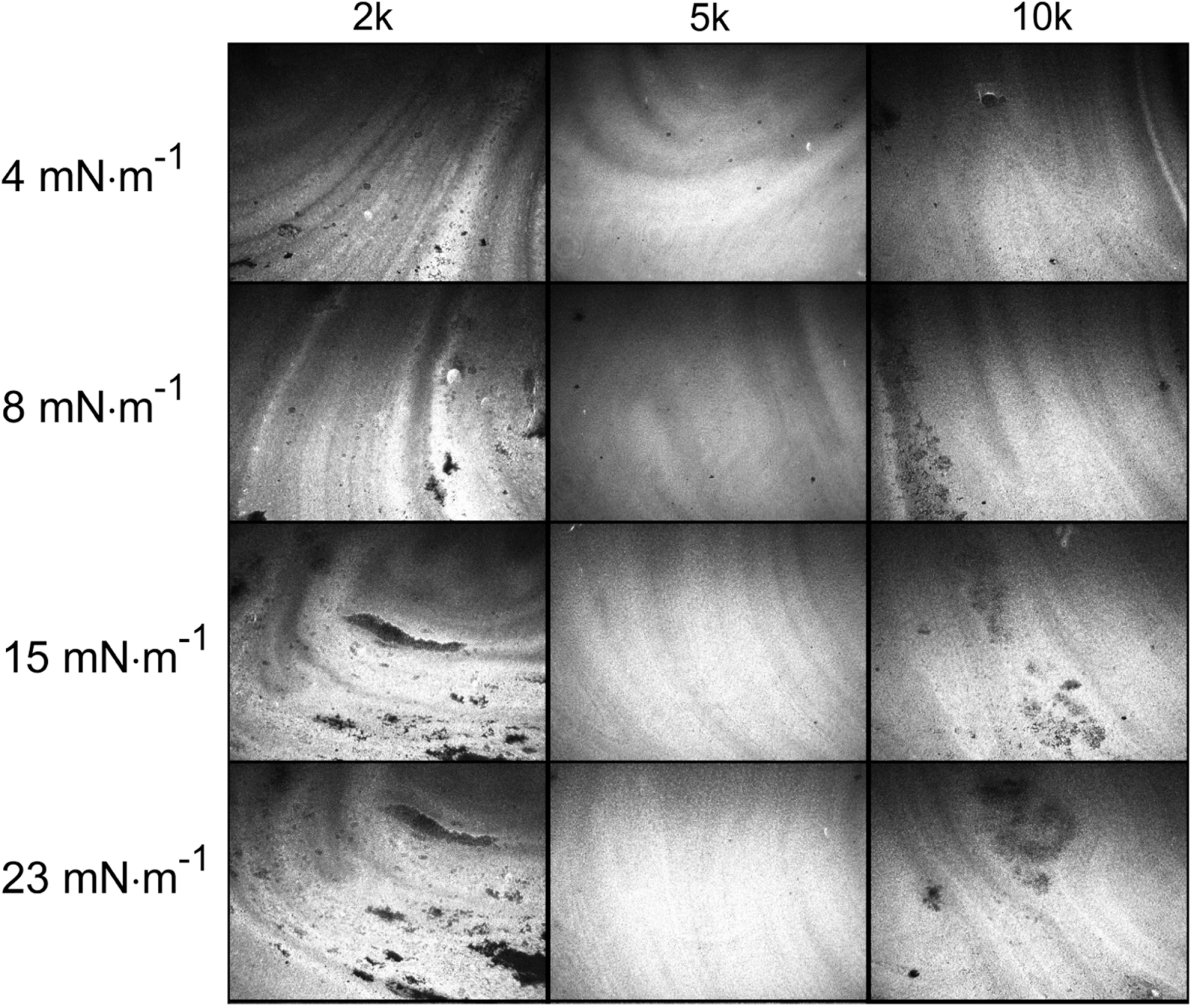
BAM images of gold nanorods for different PEGs and different surface pressures. The image width is 4000 μm.

The Langmuir monolayers were also examined by using the BAM technique (Figure 2). The obtained images provided data for the morphological analysis of the changes that occurred on the surface of the monolayer due to differences in PEG molecular weight. BAM images confirmed that each monolayer (PEG-2k, PEG-5k, and PEG-10k) formed a layer of Au-NRs while reducing the trough area. However, depending on the PEG used, the monolayers differed in structure. In the case of PEG-2k, the surface film exhibited the most heterogeneous structure throughout the compression period, owing to the numerous bands visible over the whole range of surface pressures. This may stem from the higher amount of CTAB present in the aqueous subphase because, according to our previous studies, the Au-NRs monolayer became more homogeneous with increasing surface pressure.^12^ The effect of obtaining a homogeneous monolayer was visible on the surface film containing PEG-5k. At both low and high surface pressures, similar effects were observed, indicating the presence of nonaggregated Au-NRs on the surface of the subphase. For the monolayer containing PEG-10k, agglomerates were visible with increasing surface pressure, indicating a reduced homogeneity of the surface film. Upon monolayer compression, changes were not observed in Au-NRs Langmuir monolayer color, highlighting the nonaggregated character of Au-NRs. Moreover, for all PEGs, we recorded *in situ* absorption spectra of Langmuir monolayers (results not shown). We observed the nonaggregated character of Au-NRs spectra, and the position of the longitudinal LSPR peak was linearly dependent on the surface pressure, which confirmed the uniform character of the Au-NR monolayer.

**Figure 3:**
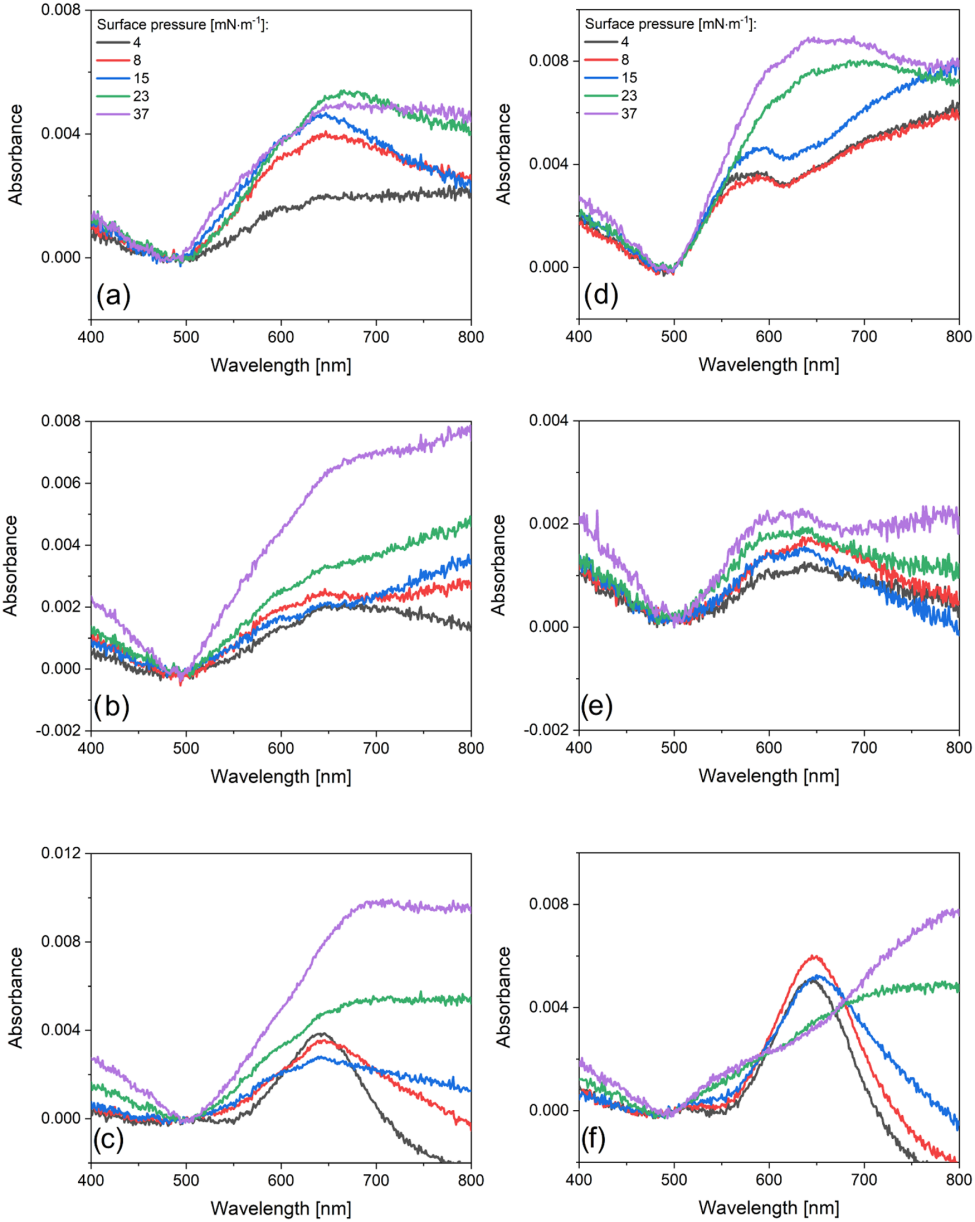
Absorption spectra of the Langmuir–Schaefer gold nanorods (a–c) and Langmuir–Blodgett (d–f) layers deposited on quartz for different PEG: PEG-2k (a, d), PEG-5k (b, e), and PEG-10k (c, f).

**Figure 4:**
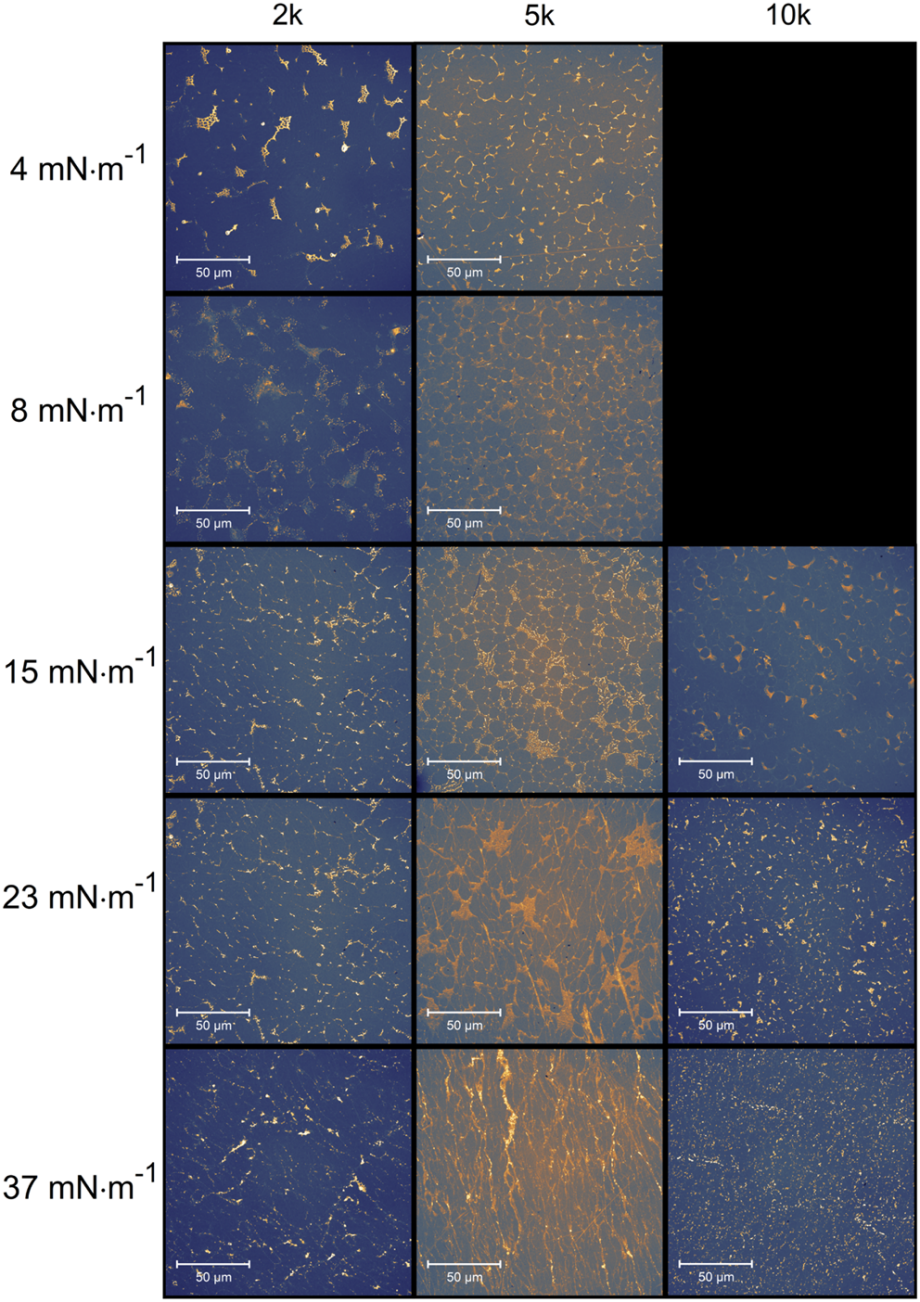
Examples of confocal microscopy images of Langmuir–Schaefer layers of gold nanorods functionalized with either 2, 5, or 10k PEG deposited on quartz substrates at different surface pressures. The longitudinal sides of the panels represent the longer side of the Langmuir trough.

**Figure 5:**
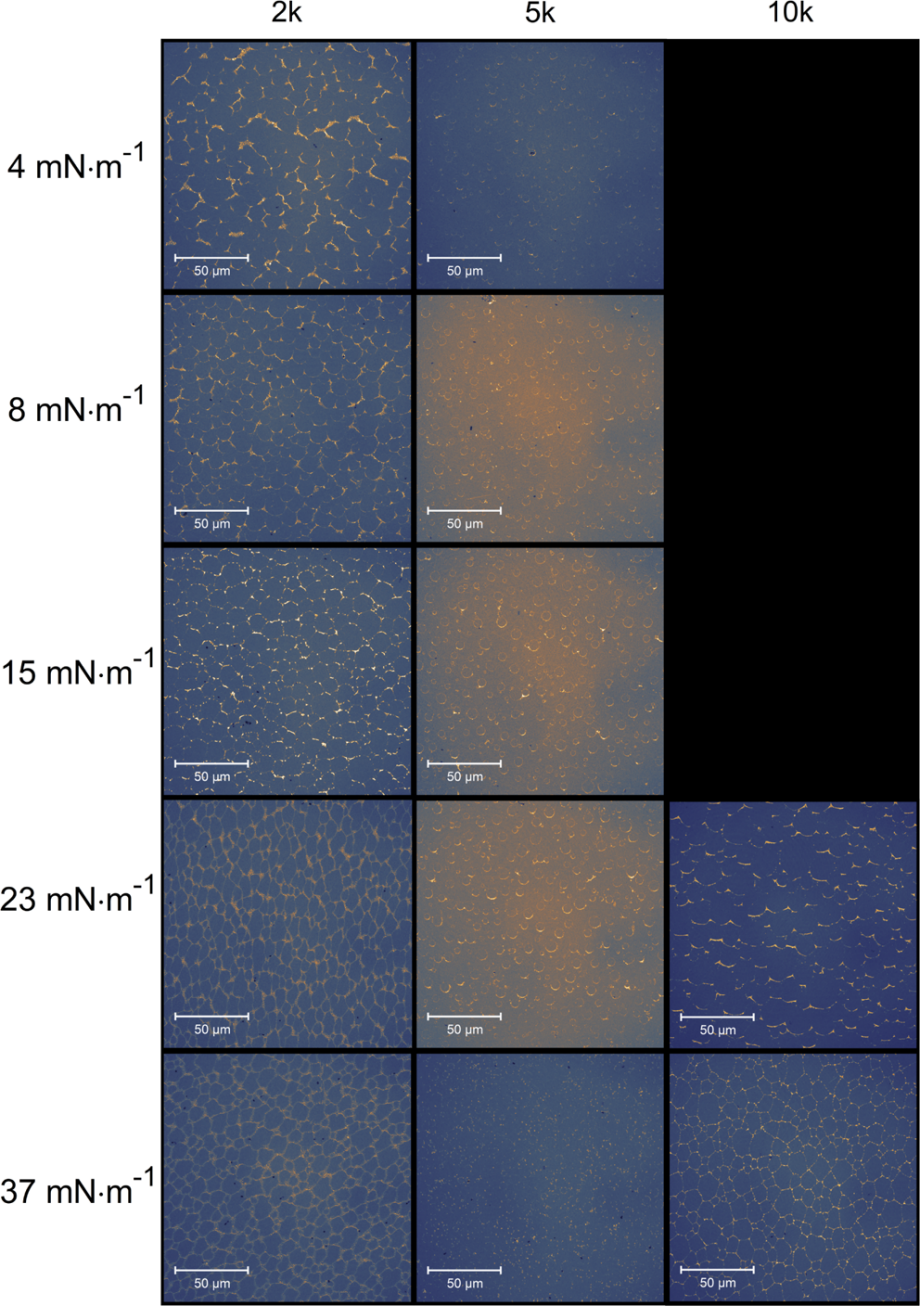
Examples of confocal microscopy images of Langmuir–Blodgett layers of gold nanorods functionalized with either 2, 5, or 10k PEG deposited on quartz substrates at different surface pressures. The longitudinal sides of the panels represent the longer side of the Langmuir trough.

**Figure 6:**
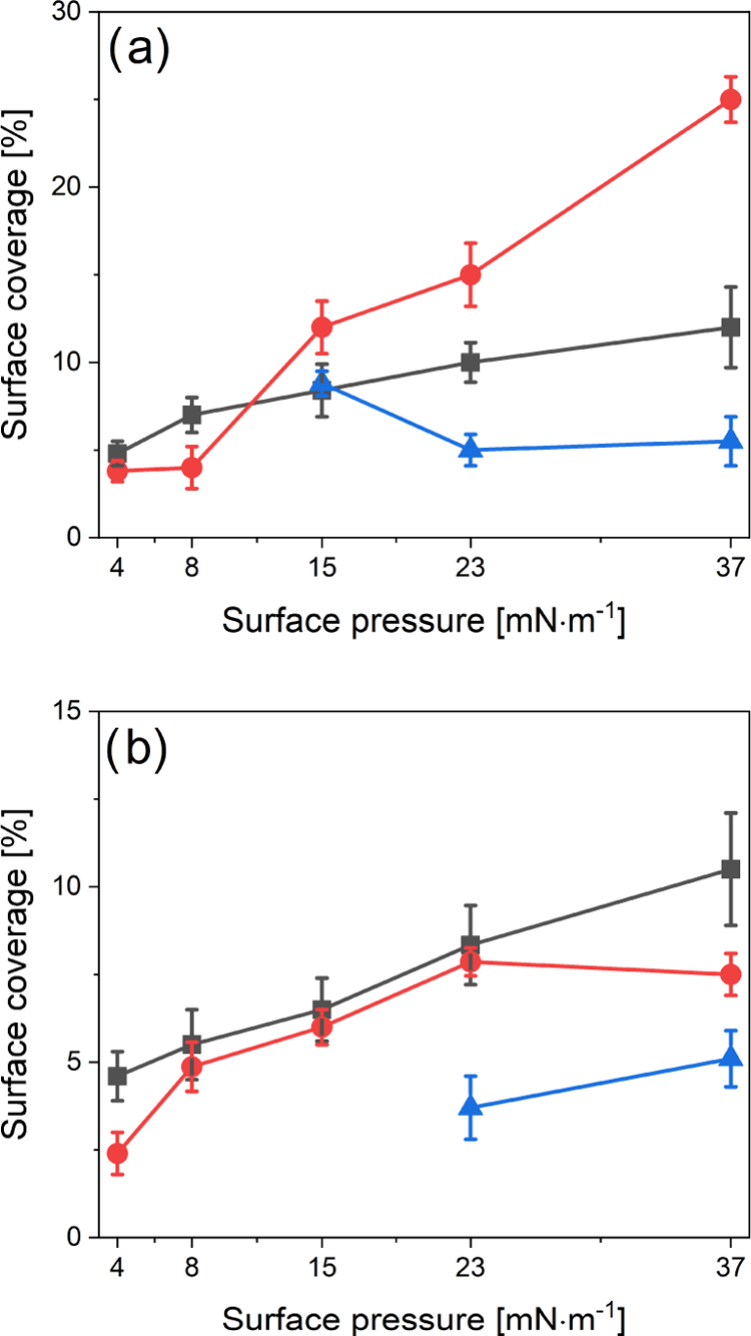
Dependence of the surface coverage of gold nanorods vs. deposition surface pressure for the Langmuir–Schaefer (a) and Langmuir–Blodgett (b) layers and different PEG: PEG-2k is a black line, PEG-5k is a red line, and PEG-10k is a blue line.

To study the Au-NRs aggregation mechanism at the interfaces induced by the PEG brush, we performed absorption spectra measurements, confocal microscopy analysis, and surface coverage studies (Figures 3–6). NPs were used to aggregate over different processes, either spontaneous or forced.^28,29^ The aggregation influenced the optical properties of individual NPs, i.e., changes in the extinction spectra observed by peak shifting or broadening. The absorption spectra of LS and LB layers shown in Figure 3 display various degrees of Au-NRs aggregation in the monolayers. Serrano-Montes et al.^30^ described the homogeneous arrangement of Au-NRs, where a gradual decrease in absorbance was observed at wavelengths above 900 nm. Thus, the spectra (Figure 3) were recorded in a similar spectral region, i.e., 400–800 nm. Moreover, only the UV–vis range of electromagnetic radiation was considered in the case of film application in surface-enhanced spectroscopies.

PEG with the longest alkyl chain (PEG-10k) and the lowest π ensured nonaggregation of Au-NRs in LS monolayers (Figure 3c). The shape of the Au-NRs spectrum was similar to that observed in the Au-NRs solution. For higher π, the main peak was red-shifted. This behavior was also observed for PEG-2k and PEG-5k. The absorption peaks were broad and red-shifted upon increasing π. In both cases, two peaks were detected at 530 and 650 nm. For PEG-5k, the third peak appeared above 800 nm. All PEGs and LS layers showed a peak at 630 nm, which suggested a red shift upon increased π. Furthermore, a different behavior was observed for LB layers (Figure 3d–f). LB deposition ensured the monomer character of Au-NRs for PEG-10k and three out of five π values (4, 8, and 15 mN·m^–1^).

The position longitudinal LSPR for nonaggregated Au-NRs was the same as for LS and LB. The highest π of 23 and 37 mN·m^–1^ displayed a peak due to the aggregation of Au-NRs above 750 nm. A similar effect was observed for LS layers and PEG-10k. Additionally, the thickest PEGs (PEG-5k and PEG-10k) did not promote the formation of nonaggregated Au-NRs layers. PEG-5k Au-NRs formed the same type of aggregate over the whole π range, with only a small increase in absorbance observed (Figure 3e). This was also the case for PEG-2k and at π ≤ 15 mN·m^–1^, while a further increase in π modified the aggregate type due to the main peak at 570 nm for lower π being red-shifted. Similar behavior was reported by Solís et al.^31^ and by Serrano-Montes et al.^30^ Solís et al.^31^ used state-of-the-art electromagnetic computation techniques to produce predictive simulations for a wide range of nanoparticle-based SERS substrates, including realistic configurations consisting of random arrangements of hundreds of nanoparticles. The authors stated that the aggregation of the particles in dimers and monolayers produced additional red shifts of the spectral features and broadening of LSPR bands caused by interparticle gaps as well as an increase in the magnitude of extinction. However, due to the complex character of the spectra, the authors did not perform any deconvolution to distinguish the basic plasmon modes. Moreover, Serrano-Montes et al.^30^ showed a significant red shift and broadening of LSPR bands due to small interparticle distances that led to plasmon coupling. The detected characteristics of the extinction spectra also occurred in cube-shaped nanoprisms NPs in polymer-grafted self-assembled layers.^32,33^

The aggregation behavior of Au-NRs at the interfaces was also examined by microscopic slide studies using confocal microscopy (Figures 4–5). The arrangement of Au-NRs in a micrometric size scale was investigated due to the aggregation behavior of Au-NRs. Moreover, the surface coverage was calculated from the obtained data with a standard deviation for both the LS and LB layers (Figure 6). For monomer Au-NRs, satisfactory images were not recorded and are indicated in Figures 4–5 as black images. According to the spectra, PEG and LS deposition layers became more aggregated. Hence, the surface coverage and density of the aggregates were higher (Figure 6). Upon increasing π to 37 mN·m^–1,^ longitudinal aggregates were formed for PEG-5k and LS layers, which was in contrast to the other layers studied. However, they did display small micrometric aggregates at π ≥ 15 mN·m^–1^. Larger but less dense structures were observed for PEG-2k and π ≤ 8 mN·m^–1^. A new type of aggregation of PEG-2k LB layers for π ≥ 15 mN·m^–1^ was clearly shown in the confocal images. Similar images were obtained for PEG-5k, while for PEG-10k and π ≥ 23 mN·m^–1^, a structural change of Au-NRs was detected as the film had a more foam-like character than that of PEG-2k. We performed SEM studies for selected Au-NR monolayers deposited at air–solid interfaces to obtain additional information about the studied systems. Results for the LB layers are shown in Figure S6, and the obtained aggregates can be seen. The aggregate formation process can be explained using two mechanisms. According to our previous studies for gold nanorods, the formation of aggregates at the microscale is influenced by the dewetting process that takes place during substrates pulling from the air–water interface,^12^ while at the nanoscale, Au-NRs tend to form two main aggregate structures. Similar results for spherical shape NPs showed different arrangements of NPs at the micro- and nanoscale.^34^ One is *end-to-end*, and the second is *side-to-side*.^35^ The two types of structures dominate the shape of the spectra shown in Figure 3 and thus produce strong plasmon coupling. It was recently shown through large-scale realistic simulation for NP-arranged systems^31^ that plasmon coupling leads to strongly confined resonances in Au-NRs aggregates.

The surface coverage of Au-NRs vs deposition surface pressure for the LS and LB layers is shown in Figure 6. The highest surface coverages were obtained for LS layers. Not all presented data points could be fitted to a linear plot. However, the dependence of PEG-2k and LB was linear for all π, whereas PEG-5k LS showed two regions for π ≤ 8 mN·m^–1^ and π > 8 mN·m^–1^. These results were consistent with the absorption spectra (Figure 3b) and confocal images of the PEG-5k LS layers. For π > 8 mN·m^–1^, a new type of aggregate was formed due to the presence of a third peak in the spectrum, which red-shifted toward above 750 nm. PEG-2k and PEG-5k LB layer’s surface coverage was the same in all π ranges within the experimental error. However, in PEG-10k, reduced surface coverage was observed, compared to PEG-2k and PEG-5k–probably due to the coexistence of aggregated (visible in confocal microscope images) and nonaggregated (invisible in confocal microscope images) structures in LB layers. The same molecular mechanism of coexistence of two phases most likely occurred for PEG-10k and LS layers. Tim et al.^12^ showed that the surface coverage did not remain as high after adding CTAB to obtain more stable Langmuir layers for all PEGs tested.

The mechanism of aggregated Au-NRs structure formation at the air–solid interfaces is a complex process. The density and optical properties of the films are influenced by many factors, mainly connected to Au-NRs synthesis and functionalization. The length of the PEG chain and its conformation at the Au-NRs surface dictated the type of aggregation. Previous studies by Rahme et al.^7^ for PEG in spherical NPs showed different behaviors of NPs as a function of PEG chain length. The number of PEG molecules grafted per NP showed that the grafting density decreased in a nonlinear trend as a function of increasing PEG length due to the increased conformational entropy and the diffusion rate of free HS-PEG, which decayed exponentially with the increase of PEG *M*_w_.^17^ PEG-functionalized Au-NRs are amphiphilic; hence, only PEG-2k Au-NRs could form stable and insoluble Langmuir monolayers in the aqueous subphase without an additional stabilizer.^12^ The coverage degree of PEG molecules on the Au-NPs surface can be estimated using thermogravimetric analysis.^7^ We tried to perform such an analysis; however, in the case of Au-NRs, where the concentration of the final product is lower than the one for Au-NPs, estimating the PEG amount at the Au surface would be practically impossible. In this case, the presented results can be explained as follows. In the case of PEG-5k and PEG-10k Au-NRs, the Langmuir monolayers were stabilized using CTAB. The additional, constant amount of CTAB (as described at the beginning of this section) was present in all Au-NRs PEGs spreading solutions because it was not completely removed upon Au-NRs centrifugation. Different conformations of PEG chains were obtained, depending on the molecular weight and PEG grafting density at the NP surface. Starting at low values of both mentioned above, the conformation from mushroom brush to brush was changed to a more dense brush.^16^ Based on the results presented in Figure 1, PEG-10k had a mushroom-brush configuration and transformed into a brush for PEG-2k due to the shifting of isotherms toward a smaller trough area. When the solid substrate was extracted from the water (in the LS or LB technique), PEG conformation controlled the aggregation process of Au-NRs at the air–solid interface. Probably, mushroom brush ensured Au-NRs in a monomer formation at low π with a tendency for stronger aggregation upon LS deposition. A denser brush with PEG chains perpendicular to the Au-NR surface ensured aggregate formation with higher packing of LS layers. On the one hand, CTAB addition allowed the generation of more stable Langmuir monolayers. On the other hand, it reduced the ability of the PEG Au-NRs to aggregate into a more complex structure. As we reported previously, stability was improved over time in Langmuir-like layers upon a small addition of surfactant.^25^ Furthermore, the dewetting process after Au-NRs deposition in such a way that the Langmuir monolayer is more stable did not affect the Au-NRs arrangement but the PEG chain conformation.

## Conclusions

The presented study investigated the formation of Langmuir–Schaefer and Langmuir–Blodgett layers consisting of PEGylated Au-NRs with different poly(ethylene glycol) alkyl chain lengths. An interesting physicochemical problem was examined by focusing on poly(ethylene glycol)’s role in forming patterns on a solid substrate. The results indicated that insoluble and stable Langmuir monolayers on the ultrapure water subphase of Au-NRs with longer poly(ethylene glycol) formed only in the presence of an additional stabilizer. This amphiphilic stabilizer was used to prevent the aggregation of Au-NRs in aqueous solutions. It has been proven that the poly(ethylene glycol) conformation influenced the shape of the compression isotherm. Hence, specific shifts during the Langmuir monolayer compression were observed. Moreover, the conformational changes in poly(ethylene glycol) induced the aggregation of Au-NRs, resulting in aggregates with specific structures. Aggregated and nonaggregated structures could be designed without requiring two or more Au-NRs functionalization steps. When the transfer surface pressure increased, the aggregates became closely packed. Their orientation was random or quasiparallel to the direction of removal of the quartz substrate from the aqueous subphase. Moreover, we demonstrated that the degree and the type of Au-NRs aggregation could be tailored depending on the molecular weight of poly(ethylene glycol) and the addition of a stabilizer. The results suggest the potential of producing patterns of hydrophilic, PEGylated Au-NRs thin layers for photonics applications, with a particular focus on optical sensing. In this application area, plasmonic substrates with the desired optical properties are beneficial for the enhanced detection of different analytes.

## Supporting Information Available

Functionalization scheme, absorption spectra, TEM image and DLS studies of gold nanorods, relaxation studies of gold nanorods Langmuir monolayer, and SEM images of Langmuir–Blodgett layers of gold nanorods (PDF)
