# Supplementary material for: The Role of the Polyethylene Glycol in the Organization of Gold Nanorods at the Air–Water and Air–Solid Interfaces
Source: Langmuir. 2024 Jul 4;40(28):14561–9. doi: 10.1021/acs.langmuir.4c01427 (PMC11256738; doi:10.1021/acs.langmuir.4c01427)
Supplement: Supplementary file 1 — la4c01427_si_001.pdf [file la4c01427_si_001.pdf]

Supplementary information

for

The Role of the Polyethylene Glycol in the Organization of Gold Nanorods at the Air-Water and the Air-Solid Interfaces

Michał Kotkowiak[a,*], Beata Tim[a], Mateusz Kotkowiak[a], Joanna Musiał[b], Paulina Błaszkiewicz[a]

[a]Faculty of Materials Engineering and Technical Physics, Poznan University of Technology, Piotrowo 3, 60-965 Poznan, Poland

[b]Department of Rare Earths, Faculty of Chemistry, Adam Mickiewicz University, 61-614 Poznan, Poland

To whom correspondence should be addressed:
*E-mail: michal.kotkowiak@put.poznan.pl (MK)
Tel.: + 48 61 665 3182

Table of contents

1. Functionalization scheme, absorption spectra, TEM image and DLS studies of gold nanorods
2. Relaxation studies of gold nanorods Langmuir monolayer
3. SEM images of Langmuir-Blodgett layers of gold nanorods

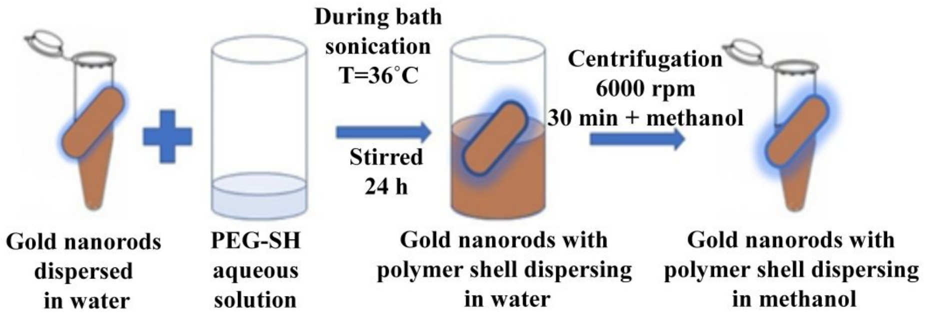

Fig. S1. Functionalization of gold nanorods by PEG coating.

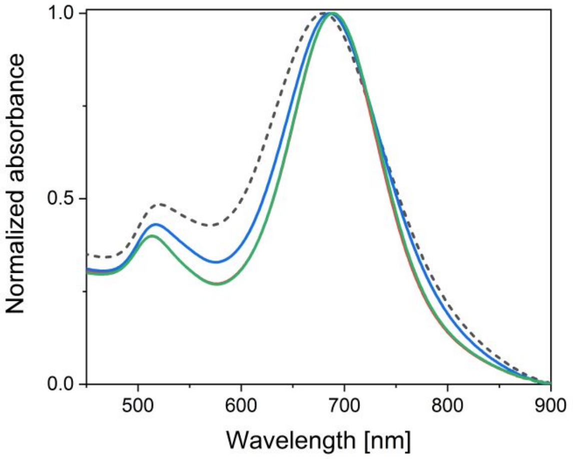

Fig. S2. Normalized absorption spectra of the gold nanorods before (dashed black line) and after PEGylation for different PEG: PEG-2k (red solid line), PEG-5k (blue), and PEG-10k (green).

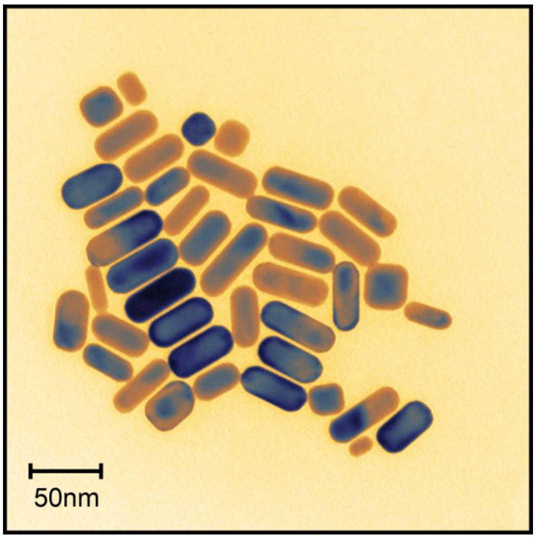

Fig. S3. Transmission electron microscopy image of the bare gold nanorods.

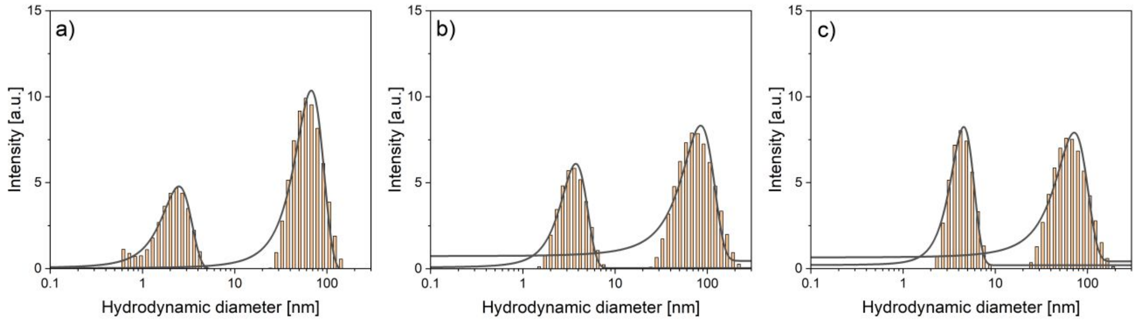

Fig. S4. Hydrodynamic diameter distribution of gold nanorods functionalized with different PEG: PEG-2k **(a)**, PEG-5k **(b)** and PEG-10k **(c)**.

Table S1. Parameters of synthesized gold nanorods functionalized with PEG

	Hydrodynamic diameter [nm]*	Zeta potential/ pH values [mV]**/[-]	LSPR localization Transverse/longitudinal [nm]
CTAB protected Au-NRs	–	34.0±0.8/7.0***	519/680
Au-NRs PEG-2k	68±2	15.0±0.4/4.8	514/688
Au-NRs PEG-5k	84±3	11.4±0.4/4.3	517/686
Au-NRs PEG-10k	72±2	-10.6±1.4/4.6	514/689

* – in methanol

** – in water/methanol mixture (9:1 v:v)

*** – in water

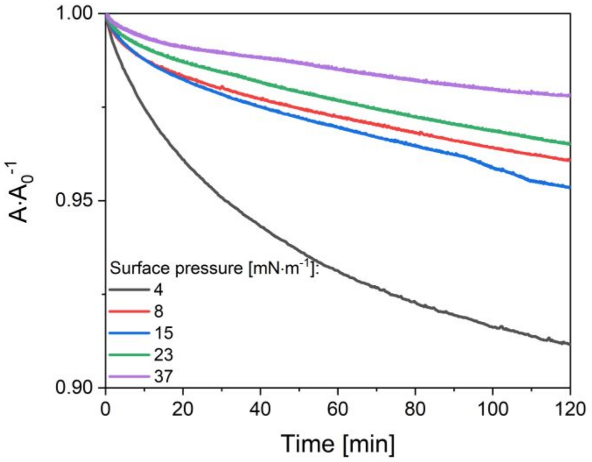

Fig. S5. Relative area changes *versus* time for a various surface pressure values of gold nanorods Langmuir monolayer functionalized with PEG-2k.

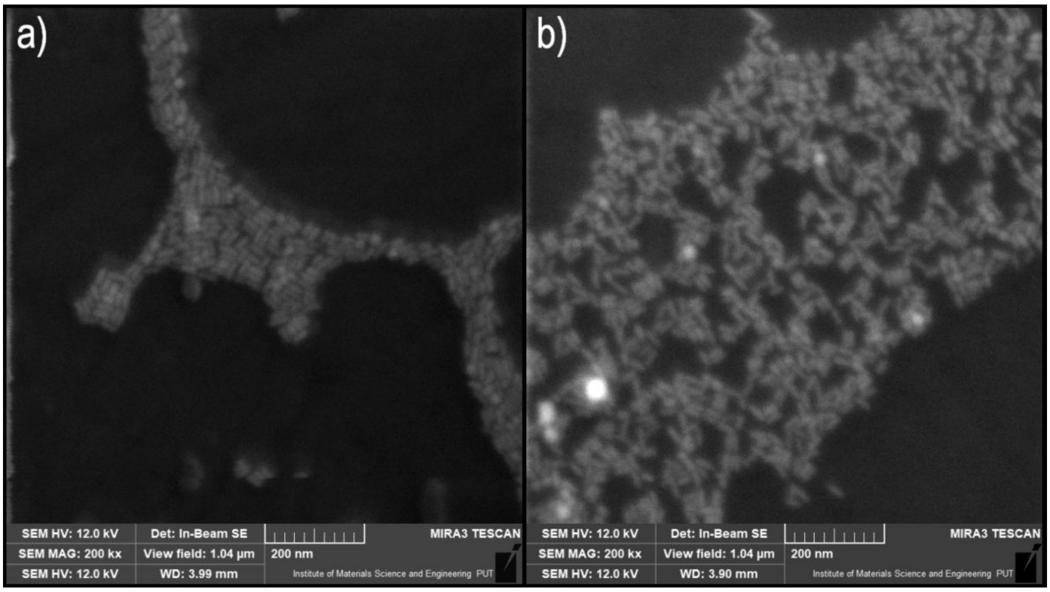

Fig. S6. Example of scanning electron microscopy images of Langmuir-Blodgett layers of gold nanorods functionalized with 2 **(a)** and **(b)** 10k-PEG, deposited on quartz substrates at surface pressures of 37 $mN \cdot m^{-1}$.
